# Two cases of long-lasting, sub-microscopic *Plasmodium malariae* infections in adults from coastal Tanzania

**DOI:** 10.1186/s12936-019-2787-x

**Published:** 2019-04-29

**Authors:** Tobias Schindler, Said Jongo, Fabian Studer, Maximilian Mpina, Grace Mwangoka, Sarah Mswata, Kamaka Ramadhani, Julian Sax, L. W. Preston Church, Thomas L. Richie, Marcel Tanner, Stephen L. Hoffman, Salim Abdulla, Claudia Daubenberger

**Affiliations:** 1 Department of Medical Parasitology and Infection Biology, Swiss Tropical and Public Health Institute, Basel, Switzerland; 2 University of Basel, Basel, Switzerland; 3 Ifakara Health Institute, Bagamoyo Research and Training Centre, Bagamoyo, United Republic of Tanzania; 4 Sanaria Inc., Rockville, MD USA

**Keywords:** *Plasmodium malariae*, Asymptomatic malaria, Quantitative polymerase chain reaction (qPCR)

## Abstract

**Background:**

Malaria is endemic in Tanzania with majority of clinical cases caused by *Plasmodium falciparum*. Additionally, *Plasmodium malariae* and *Plasmodium ovale* spp. are also present and clinical manifestations caused by these infections are not well described. Clinical episodes caused by *P. malariae* infections are often characterized by a relatively mild illness with a low number of parasites, which can persist for long periods. In this report, two cases of *P. malariae* infections that were identified during a clinical trial evaluating the *P. falciparum* malaria vaccine candidate, PfSPZ Vaccine are described. The two participants were followed up and monitored for clinical and laboratory parameters to assess vaccine safety providing the opportunity to study clinical manifestations of *P. malariae* over 4 months.

**Case presentation:**

Two young, healthy Tanzanian men infected with low density asexual blood stage *P. malariae* diagnosed by quantitative polymerase chain reaction (qPCR) are described. Retrospective analysis of collected and stored blood samples revealed that the two volunteers had constant asexual blood stage parasitaemia for more than 4 months. During the 132 days of infection, the volunteers’ vital signs, body temperature and serum biochemistry all remained within normal ranges. Haematological abnormalities, which were transiently outside normal ranges, were regarded as not clinically significant. During this time period, four consecutive evaluations of blood samples by thick blood smear microscopy conducted by an experienced microscopist were all negative, indicating the presence of low-density sub-microscopic infections.

**Conclusions:**

The two cases of *P. malariae* infections presented here confirm the ability of this *Plasmodium* species to persist at low density in the human host for extended time periods without causing clinical symptoms. The presented data also demonstrate that clinical study sites in malaria endemic regions need to have a strong malaria diagnostic infrastructure, including the ability of capturing sub-microscopic parasitaemia and differentiation of *Plasmodium* species.

*Trial registration* ClinicalTrials.gov: NCT02613520, https://clinicaltrials.gov/ct2/show/NCT02613520, Registered: November 24th 2015, Enrolment of the first participant to the trial: December 15th 2015, Trial was registered before the first participant was enrolled

## Background

Malaria is endemic in Tanzania with more than 90% of the population at risk and 5.6 million cases reported by the public health sector in 2017 [1]. While *Plasmodium falciparum* is the dominant malaria species responsible for majority of infections and deaths, other *Plasmodium* species are also endemic in Tanzania. David Clyde, who served as the director of the Malaria Service of the East African Malaria Institute at Amani, described the occurrence of four human malaria species, including *P. falciparum*, *Plasmodium vivax*, *Plasmodium ovale* spp. and *Plasmodium malariae* in his 1967 book “*Malaria in Tanzania*” [2]. *Plasmodium vivax* was attributed to importation by Indian immigrants during the first world war and since 1917 this influx has virtually ceased. *Plasmodium malariae* was observed in 10–20% of malaria infections, mainly as co-infections with *P. falciparum* and during childhood [2]. More recently, a microscopy-based cross-sectional survey conducted in the Tanga region of coastal Tanzania found very few infections with *P. malariae* (0.3%) or *P. ovale* spp. (0.1%) [3]. Data collected in coastal Tanzania, confirm these low numbers of non-*P. falciparum Plasmodium* infections. Diagnosis by qPCR revealed low prevalence for *P. malariae* (0.7–5.8%) and *P. ovale* spp. (0.9–1.1%) among asymptomatic school children (Schindler et al., unpublished data). Since microscopic diagnosis of *P. malariae* asexual blood stage parasites is hampered by the low parasitaemia and morphological similarities to *P. falciparum*, molecular based, highly sensitive diagnostic methods are needed to establish the true prevalence of this parasite in the population [4]. Lack of sensitive *P. malariae* diagnosis methods applicable in the field and the research focus on *P. falciparum* has resulted in significant knowledge gaps regarding spectrum of potential clinical manifestations and burden of *P. malariae* infections [5].

It is well established that *P. malariae* is widespread throughout sub-Saharan Africa, South East Asia and Latin America and the biology of *P. malariae* was reviewed by Collins et al. [6]. Treatment of syphilis by controlled infections with *P. malariae* provided valuable insight into human-parasite interactions. The red blood cell cycle lasts 72 h with an average of 8 merozoites released per schizont and the parasite prefers to infect and develop in older erythrocytes. So far, no evidence for a dormant liver stage as described in *P. vivax* and *P. ovale* spp. has been observed. Faster acquisition of immunity against *P. malariae* compared to immune responses against *P. falciparum* has been described [6].

Clinical episodes of *P. malariae* infections are characterized by a mild illness caused by low numbers of parasites which can persist for extremely long periods, often for years or even decades [6]. There are reports of cases of *P. malariae* caused illness from Greece [7] and Trinidad and Tobago [8] decades after eradication of malaria from these regions. Chronic *P. malariae* infections have been considered a major cause of the nephrotic syndrome in the past, although the incidence of *P. malaria*-associated nephrotic syndrome has been dramatically reduced in recent decades [7–9]. Recently, it was demonstrated that the controlled infection of two volunteers with cryopreserved *P. malariae*-infected erythrocytes was well tolerated and no severe or serious adverse effect, or biochemical abnormalities were observed [10].

The clinical research facility of the Ifakara Health Institute in Bagamoyo, Tanzania, conducts clinical trials evaluating efficacy of experimental malaria vaccines in the target population [11–13]. A controlled human malaria infection (CHMI) model has been successfully established since 2012 [14]. As part of these clinical trials, participants are closely monitored to identify any abnormal clinical or laboratory parameters in order to evaluate vaccine safety and tolerability. Regularly, volunteers are screened for *Plasmodium* spp. parasites in blood using thick blood smear microscopy as well as quantitative polymerase chain reaction (qPCR). The volunteers described in this report participated in a study evaluating the safety and efficacy of immunization with Sanaria^®^ PfSPZ Vaccine composed of radiation attenuated, aseptic, purified, cryopreserved *P. falciparum* sporozoites (PfSPZ) [11, 15–19] which was conducted between 2015 and 2016 (clinicaltrials.gov: NCT02613520) [20]. The clinical cases of two young men infected with asexual blood stage *P. malariae* as diagnosed by qPCR are described. These volunteers were followed closely for 4 months during the clinical trial.

## Case presentation

**Fig. 1:**
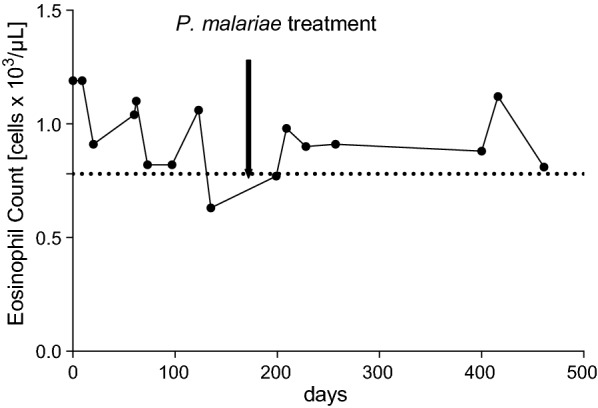
Elevated eosinophil counts for volunteer 2 over a time period of more than 400 days. Eosinophil counts of volunteer 2 covering all visits, from study enrolment to completion, are shown. The dashed line represents the upper limit of the normal range (0.78 × 10^3^ cells/µL)

Two male residents of Bagamoyo, 20 and 22 years of age, were enrolled into the clinical trial based on predefined exclusion and inclusion criteria as outlined in the clinical trial protocol. A review of the medical history, physical examination, vital signs (pulse, blood pressure, and respiratory rate), and ECG did not reveal any abnormalities. At screening, the volunteers had negative serologies for human immunodeficiency virus (HIV), hepatitis B virus (HBV), and hepatitis C virus (HCV). A single stool sample collected at study enrolment was negative for intestinal helminths and no *Schistosoma haematobium* eggs were detected in urine. No blood biochemistry abnormalities were detected, which included alanine aminotransferase (ALT), aspartate aminotransferase (AST), total bilirubin (BIL), and creatinine (CRE). The urine analysis using a 13-parameter dipstick (Combina 13 test strips, HUMAN Diagnostics, Germany) was negative at enrolment. A complete blood count (CBC) was conducted at screening and showed normal haematological parameters for volunteer 1, while volunteer 2 had elevated eosinophil counts (1.19 × 10^3^/µL compared to the pre-defined upper normal range of 0.78 × 10^3^ cells/µL). During the 16 weeks of immunization (3 doses of PfSPZ Vaccine at 8-week intervals), the volunteers’ health status was closely monitored. Every 8 weeks at pre-defined visits blood was drawn and any deviations from reference laboratory values were reported. Vital signs, body temperature and biochemistry remained within normal ranges when assessed four times during the follow up period. Haematological parameters which were transiently outside normal ranges in both volunteers were regarded as not clinically significant (Tables 1, 2). Noteworthy, for volunteer 2 the eosinophil counts remained consistently elevated until the end of the study (Fig. 1). Both volunteers tested once positive for low grade proteinuria, during the study by urine dipstick (Tables 1, 2).

**Table 1:** Overview of clinical and parasitological parameters assessed for volunteer 1

	Study enrolment	Routine visit 1	Routine visit 2	qPCR results reported	Post-treatment visit 1	Post-treatment visit 2
Timeline						
Date	22/12/2015	13/02/2016	13/04/2016	28/04/2016	06/05/2016	01/07/2016
Days respective to enrolment into clinical trial	0	53	113	128	136	192
Clinical evaluation						
Body temperature	36.6 °C	36.2 °C	36.8 °C	36.6 °C	–	37.2 °C
Vital signs including pulse, blood pressure and respiration rate	NAD	NAD	NAD	NAD	–	NAD
Laboratory evaluation						
Biochemistry including ALT, AST	NAD, also included BIL and CRE at enrolment	NAD	NAD	NAD	–	NAD
Complete blood count includes RBC, HGB, HCT, MCV, MCH, MCHC, PLT, WBC with differential	NAD	NEUT ↑ (3.57 × 10^3^/µL, 72.2%) LYMP ↓ (0.70 × 10^3^/µL, 14.1%)	MCHC ↓ (29.7 g/dL)	NAD	–	NAD
Urine analysis using dipstick that includes bilirubin, ketone, creatinine, hematuria, proteinuria, albumin, nitrite, leucocytes, glucose, specific gravity, pH, vitamin C	NAD	NAD	Proteinuria (1+)	NAD	–	NAD
Serology (HIV, HBV, HCV)	Negative	–	–	–	–	–
Screening for intestinal helminths and schistosomiasis	Negative	–	–	–	–	–
Malaria diagnostics						
Thick blood smear microscopy	Negative	Negative	Negative	Negative	Negative	Negative
Screening and species identification qPCR assays	Screening qPCR: *Cq of 36.03* ID-qPCR: *P. malariae*	Screening qPCR: *Cq of 35.72* ID-qPCR: *P. malariae*	Screening qPCR: *Cq of 37.69* ID-qPCR: *neg*^a^	Screening qPCR: *Cq of 34.47* ID-qPCR: *P. malariae*	Screening qPCR: *neg*	Screening qPCR: *neg*
Drug treatment						
Prescription of drugs throughout the clinical trial	–	–	–	Start of artesunate-amodiaquine based treatment on 02/05/2016	–	–

*NAD* no abnormalities detected

^a^*Plasmodium* species identification qPCR assay was negative due to drop of *P. malariae* parasitemia (Cq value for 18S *Plasmodium* spp. screening assay was 37.69)

**Table 2:** Overview of clinical and parasitological parameters assessed for volunteer 2

	Study enrolment	Routine visit 1	Routine visit 2	qPCR results reported	Post-treatment visit 1	Post-treatment visit 2
Timeline						
Date	22/12/2015	13/02/2016	13/04/2016	28/04/2016	06/05/2016	01/07/2016
Days respective to enrolment into clinical trial	0	53	113	128	136	192
Clinical evaluation						
Body temperature	36.5 °C	36.8 °C	36.7 °C	36.6 °C	–	36.4 °C
Vital signs including pulse, blood pressure and respiration rate	NAD	NAD	NAD	NAD	–	NAD
Laboratory evaluation						
Biochemistry including ALT, AST	NAD, also included BIL and CRE at enrolment	NAD	NAD	NAD	–	NAD
Complete blood count includes RBC, HGB, HCT, MCV, MCH, MCHC, PLT, WBC with differential	EO ↑ (1.19 × 10^3^/µL, 18.1%)	EO ↑ (1.04 × 10^3^/µL, 17.2%)	EO ↑ (1.06 × 10^3^/µL, 12.0%) RDW-SD ↓ (34.4 fL)	EO ↑ (0.63 × 10^3^/µL, 6.5%) WBC ↑ (9.65 × 10^3^/µL) NEUT ↑ (6.07 × 10^3^/µL, 62.9%)	–	EO ↑ (0.77 × 10^3^/µL, 12.0%) RDW-SD ↓ (33.6 fL)
Urine analysis using dipstick that includes bilirubin, ketone, creatinine, hematuria, proteinuria, albumin, nitrite, leucocytes, glucose, specific gravity, pH, vitamin C	NAD	NAD	NAD	NAD	–	Proteinuria (trace)
Serology (HIV, HBV, HCV)	Negative	–	–	–	–	–
Screening for intestinal helminths and schistosomiasis	Negative	–	–	–	–	–
Malaria diagnostics						
Thick blood smear microscopy	Negative	Negative	Negative	Negative	Negative	Negative
Screening and species identification qPCR assays	Screening qPCR: Cq of 31.62 ID-qPCR: *P. malariae*	Screening qPCR: Cq of 32.78 ID-qPCR: *P. malariae*	Screening qPCR: Cq of 33.44 ID-qPCR: *P. malariae*	Screening qPCR: Cq of 34.47 ID-qPCR: *P. malariae*	Screening qPCR: *neg*	Screening qPCR: *neg*
Drug treatment						
Prescription of drugs throughout the clinical trial	–	–	–	Start of artesunate-amodiaquine based treatment on 02/05/2016	–	–

*NAD* no abnormalities detected

After concluding three PfSPZ Vaccine immunizations, and before vaccine efficacy was assessed by CHMI, the study protocol required screening of whole blood by qPCR to detect sub-microscopic malaria parasitaemia, so that these volunteers could be treated accordingly before participation in CHMI. During this routine visit, qPCR was conducted with fresh blood samples and it was discovered that two volunteers were infected with *Plasmodium* spp. parasites [21]. Based on *P. malariae* species-specific qPCR [22] and conventional nested PCR [23], *P. malariae* infections were confirmed. The presence of *P. falciparum* [24, 25], *P. ovale* spp. [26], and *P. vivax* [25] was excluded by qPCR and conventional PCR [23].

Treatment with 3 doses of artesunate/amodiaquine (200/540 mg) daily for 3 days was initiated, and complete parasite clearance was confirmed by qPCR 4 days later. Both volunteers then underwent CHMI and remained in the clinical trial until study completion. Within the following 296 days until the completion of the clinical trial, no recurrent (recrudescence or new infection) *P. malariae* parasitaemia was observed. Both volunteers were negative for *P. falciparum* after the first CHMI, and both became positive for *P. falciparum* after a second CHMI at 40 weeks after the last immunization and were successfully treated with artemether/lumefantrine.

**Fig. 2:**
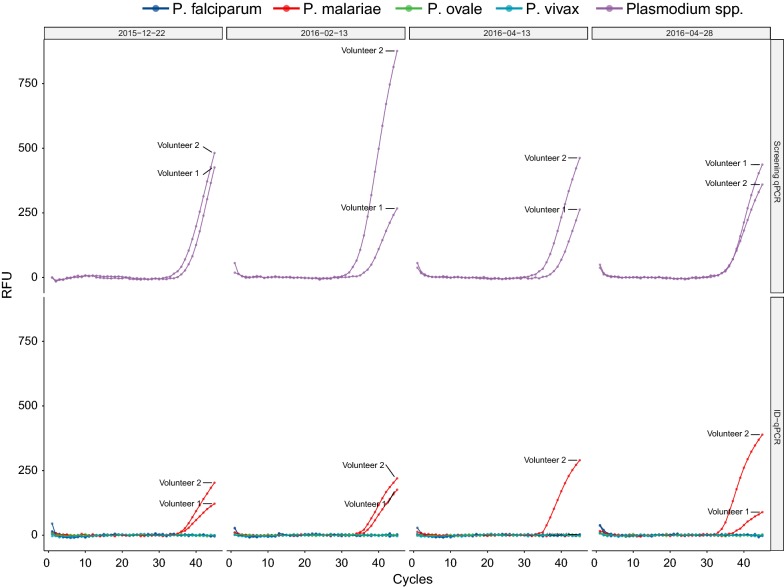
qPCR data for the *Plasmodium* spp. screening and *Plasmodium* species identification assays. The upper panel shows the amplification curves for the *Plasmodium* spp. target of the screening assay. The lower panel shows the amplification for the four *Plasmodium* species-specific targets. All samples were run in triplicates and DNA from *P. falciparum*, *P. malariae*, *P. ovale wallikeri*, *P. ovale curtisi* and *P. vivax* were included as positive controls during the qPCR

Blood samples collected during the clinical trial and stored frozen were analysed retrospectively by qPCR to determine the time point of *P. malariae* infection. It turned out that both volunteers had *P. malariae* parasitaemia at enrolment into the clinical trial. Both volunteers remained positive throughout the vaccination period. *Plasmodium malariae* parasites were detectable at four out of four clinical visits, namely at day 0, 53, 113 and 128 of study and malaria treatment took place 132 days after the first detection of the *P. malariae* infections (Fig. 2). Evaluation of four blood samples collected at the same days by thick blood smear microscopy and conducted by an experienced microscopist was reported as negative. Thick blood smear preparation and reading was performed according to our standard operating procedure followed during CHMI studies [14]. The negative microscopy results and the high Cq values (median of 34.1 with a range of 31.6–37.7) obtained by the *Plasmodium* spp. qPCR assay indicate that the parasitaemia levels were low. When compared to qPCR based detection of *P. falciparum* 18S gene, these Cq values would correspond to a parasitaemia between 1 and 10 *P. malariae* parasites per µL blood [27].

## Discussion and conclusion

The two *P. malariae* cases presented here confirm the ability of this *Plasmodium* species to persist at low density in the human host for extended time periods without causing clinical symptoms or signs. Both were detected in clinically healthy, young men participating in a clinical trial of PfSPZ Vaccine. No abnormalities in vital signs, alanine aminotransferase, aspartate aminotransferase, total bilirubin, and creatinine serum levels were detected. Except for a one-time low-level proteinuria, urine analysis parameters measured by dipstick remained within physiological ranges and there was no indication of impaired renal function in these two volunteers. Volunteer 2 did have mildly elevated eosinophil counts throughout the entire course of the clinical trial. These levels were not affected by the treatment of the *P. malariae* infection and may have reflected an ongoing intestinal helminth infection that was too low to be detected by a single stool examination. All other haematological abnormalities were of temporary nature and considered to be not clinically significant. Interestingly, the *P. malariae* parasitaemia levels were not affected by the three rounds of PfSPZ Vaccine immunizations. This might be due the mode-of-action of the vaccine which is thought to act against the liver-stage of the parasite.

The data presented in this report demonstrates that study sites in malaria endemic regions conducting clinical trials should develop on site malaria diagnostic infrastructure, which includes the detection of low-density asexual blood stage parasitaemia and identification of different *Plasmodium* species. Eventually, if the goal of malaria elimination is pursued vigorously, the implementation of highly sensitive diagnostic methods to detect asymptomatic, low-density *P. malariae* infections need to be included into the malaria elimination agenda.

## Abbreviations

PfSPZ: *P. falciparum* sporozoites
CHMI: controlled human malaria infections
HIV: human immunodeficiency virus
HBV: hepatitis B virus
HCV: hepatitis C virus
ALT: alanine aminotransferase
AST: aspartate aminotransferase
BIL: total bilirubin
CRE: creatinine
CBC: complete blood count
RBC: red blood cells
HGB: haemoglobin
HCT: haematocrit
MCV: mean corpuscular volume
MCH: mean corpuscular haemoglobin
MCHC: mean corpuscular haemoglobin concentration
PLT: platelets
WBC: white blood cells
NEUT: neutrophils
LYMP: lymphocytes
EO: eosinophils
RDW-SD: red cell distribution width
TBS: thick blood smear
qPCR: quantitative polymerase chain reaction
Cq: quantification cycles of qPCR reaction
ID-qPCR: *Plasmodium* species identification qPCR assay
NAD: no abnormalities detected
